# Editorial: Immune Checkpoint Molecules and Cancer Immunotherapy

**DOI:** 10.3389/fimmu.2018.02878

**Published:** 2018-12-05

**Authors:** Alexandr V. Bazhin, Amedeo Amedei, Svetlana Karakhanova

**Affiliations:** ^1^Department of General, Visceral, and Transplantation Surgery, Ludwig-Maximilians University, Munich, Germany; ^2^Partner Site Munich, German Cancer Consortium (DKTK), Munich, Germany; ^3^Department of Experimental and Clinical Medicine, University of Florence, Florence, Italy; ^4^Neuromusculoskeletal Department (Interdisciplinary Internal Medicine), Azienda Ospedaliera Universitaria Careggi, Florence, Italy; ^5^Section Surgical Research, University of Heidelberg, Heidelberg, Germany; ^6^Department of General, Visceral and Transplantation Surgery, University of Heidelberg, Heidelberg, Germany

**Keywords:** immune checkpoint molecules, cancer immunotherapy, CTLA-4 (CD152), PD-1 (CD279), PDL1

On October 2^nd^, we experienced with great enthusiasm that the 2018 Nobel Prize in the Medicine and Physiology goes to the fathers of check-point molecules CTLA-4 and PD-1 James P. Allison and Tasuku Honjo “for their discovery of cancer therapy by inhibition of negative immune regulation”(1, 2). This Nobel Prize assignment was a great note for us as guest editors of the special issue “Immune Checkpoint Molecules and Cancer Immunotherapy.” Hence, this topic is not just in vogue but represents an enormous important field of translational cancer immunology. The discovery of the checkpoint molecules CTLA-4 and PD-1 stimulated pharma industry to develop specific inhibitors for cancer treatments, encouraging many scientists and clinicians to further explore this field (3, 4). We have witnessed a real success! Especially about the malignant melanoma, where during more than 30 years we saw no progress in the treatment of this tremendous disease (5–7). Meanwhile, inhibitors of checkpoint molecules and their receptors are approved for the treatment of different malignancies and there are impressive case reports of patients (8).

But the way is still stony. During the experience with immunotherapeutic drugs based on targeting of immune checkpoint molecules, many questions, and problems arose. First, not all cancer types as well as not all patients, tested in immunotherapeutic clinical trials, were sensitive to the treatment. Second, it is still not clear how we can monitor the therapy successes. Are there some biomarkers to predict the response to therapy? How can the expression of these immune checkpoint molecules be modulated or influenced? What is the impact of combination of the immune checkpoint molecule therapy with a conventional cancer treatment? Undoubtedly, these and other questions require further intensive research. Therefore, we announced last year this special issue by Frontiers Immunology. The main aim was to collect novel findings from scientists and clinicians involved in basic research on immune checkpoints as well as in translational studies investigating the use of checkpoint inhibitors in immunotherapy in experimental settings.

As mentioned before, not all cancer types tested in immunotherapeutic trials with checkpoint molecule inhibitors had a benefit from such a therapy. One of these exceptions is the pancreatic adenocarcinoma (PDAC). This extremely severe cancer did not respond to the immune checkpoint inhibition treatment. Kabacaoglu et al. attempted to elucidate this problem in the review “*Immune Checkpoint Inhibition for Pancreatic Ductal Adenocarcinoma: Current Limitations and Future Options.”* The authors shed light on the immune escape mechanisms allowing PDAC to avoid the effect of immune checkpoint inhibition. Further more they discussed possibilities to potentially improve outcome of such immunotherapy in PDAC. In this line, we are pleased to introduce results of the original research of Rataj et al. “*PD1-CD28 Fusion Protein Enables CD4*+ *T Cell Help for Adoptive T Cell Therapy in Models of Pancreatic Cancer and Non-hodgkin Lymphoma,”* where intriguing data showing a potential for such fusion to overcome the immune suppression due to PD1-PD-L1 axe. Since the authors used two very different cancer models, the results of this study represent a generalized significance.

Importance of crosstalk between immune checkpoint molecules and cellular immunosuppression gets as well increasingly more consideration. About that, the review of Weber et al. “*Myeloid-Derived Suppressor Cells Hinder the Anti-Cancer Activity of Immune Checkpoint Inhibitors”* shed light on a specific immunosuppressive cell population—myeloid-derived suppressor cells, which appear to affect the suppressive potential of immune checkpoint molecules. The authors profoundly discussed the possibility to combine targeting of both immunosuppressive components. This could be valid not only for the malignant melanoma, but also in other cancers, especially in the prostate adenocarcinoma which is highlighted in the review of Elia et al. “*Immune Checkpoint-Mediated Interactions Between Cancer and Immune Cells in Prostate Adenocarcinoma and Melanoma.”*

What is new about biomarkers of response to therapy with inhibitors of the immune checkpoint molecules? Kristina Buder-Bakhaya and Jessica Hassel from Heidelberg dealt very intensely with this topic in the review “*Biomarkers for Clinical Benefit of Immune Checkpoint Inhibitor Treatment – A Review From the Melanoma Perspective and Beyond.”* The authors thoroughly aggregated the current status of prognostic and predictive biomarkers, used in immune checkpoint inhibition for melanoma and other malignances. They concluded that several possible biomarkers for response to such therapy are now available and should be validated in large clinical trials. Some unexpected relations to the regulatory molecules came as well out in this issue. De Assis et al. could trace a link between circadian clock genes (!) and the aforesaid molecules in their report “*Expression of the Circadian Clock gene BMAL1 Positively Correlates With Antitumor Immunity and Patient Survival in Metastatic Melanoma.”* Besides, the gene BMAL1 was found to be associated with an increase in the antitumor immune response but also with the clinical benefit for melanoma patients treated with checkpoint molecule inhibitors; in other words it could be a potential treatment biomarker. What is beyond? In this article collection we are pleased to welcome three original studies, which enlarge the circle of potential biomarkers also apart from malignant melanoma. An interesting report “*Indoleamine 2,3-Dioxygenase Expression Pattern in the Tumor Microenvironment Predicts Clinical Outcome in early Stage cervical Cancer”* was received from the group of Heeren et al. from Amsterdam. The authors claimed that indoleamine dioxygenase can be recognized as a real immune checkpoint molecule. Moreover, they conveniencely demonstrated that a marginal expression of this enzyme predicts a favorable outcome for patients with cervical cancer, making this protein a potential inhibitory target as well as a prognostic biomarker. Another impressive study came from Manjarrez-Orduño et al. “*Circulating T Cell Subpopulation Correlate With Immune Responses at the Tumor Site and Clinical Response to PD-1 Inhibition in Non-Small Cell Lung Cancer.”* The title of the paper is self-describing and this work opens new avenues in the field of potential blood biomarkers. In addition to that, head and neck cancer is represented in this special issue. A case report from Qatar by Merhi et al. “*Squamous Cell Carcinomas of the Head and Neck Cancer Response to Programmed Cell Death Protein-1 Targeting and Differential Markers”* introduces a patient suffering from this cancer, who was treated with Nivolumab. The authors indicated that in this case a defined cytokine/chemokine profile might be useful for identifying a response to PD-1 inhibition.

The next series of papers is devoted to the matter of regulation of checkpoint molecule expression. An amiable perspective on the post-transcriptional regulation of CD73/NT5E is delineated by the group of Kordaß et al. from DKFZ Heidelberg in the paper “*Controlling the Immune Suppression: Transcription Factors and MicroRNAs Regulating CD73/NT5E.”* They reviewed all contemporary literature concerning this point and highlighted the significance of miRNA involved in the regulation of this checkpoint molecule expression. With respect to transcription factors, Bhat et al. from India showed in their paper “*Checkpoint Blockade Rescues the Repressive Effect of Histone Deacetylases Inhibitors on* γδ *T cell Function”* that Eomes and T-bet could be potential regulators of PD-1 expression. The regulation of PD-L1 expression was assessed in the work of Bazhin et al. “*Interferon-*α *Up-Regulates the Expression of PD-L1 Molecules on Immune Cells Through STAT3 and p38 Signaling.”* The authors observed that the type I interferon is indeed involved in the regulation of PD-L1 expression through the above-mentioned transcription factors. A very unorthodox point of view presented by Wang et al. devoted to the gut microbiota in context of immune checkpoint molecules. In their review “*Modulation of Gut Microbiota: A Novel Paradigm of Enhancing the Efficacy of Programmed death-1 and Programmed death Ligand-1 Blockade Therapy”* the authors thoughtfully discussed the influence of gut microbiota on blocking of PD1-PD-L1 axis.

The authors leaded by Yan et al. were concerned with the problems of combining immune checkpoint inhibition with conventional tumor therapy in the manuscript “*Combining Immune Checkpoint Inhibitors With Conventional Cancer Therapy.”* They reviewed current literature analyzing the impact of chemo-, radio-, and target therapies on therapeutic effects of immune checkpoint inhibition and discussed the current and the future clinical applications of such combination. Finally, Shevchenko and Bazhin pointed in their work to an importance of discovery of new potential checkpoints molecules. They payed attention in their mini-review “*Metabolic Checkpoints: Novel Avenues for Immunotherapy of Cancer”* to so-called metabolic checkpoint molecules which could be the “new era” of the cancer immunotherapy with checkpoint inhibition.

Summarizing, the wide spectrum of reviews and original papers from this issue provides an insight into new research directions linked to an extremely important topic-immune checkpoint molecules in context of cancer immunotherapy. We wish all readers of this special issue to have an exciting time to take a close look into a subject of this compendium.

## Author Contributions

All authors listed have made a substantial, direct and intellectual contribution to the work, and approved it for publication.

### Conflict of Interest Statement

The authors declare that the research was conducted in the absence of any commercial or financial relationships that could be construed as a potential conflict of interest.
